# Vitamin D deficiency promoting non-24 h sleep–wake disorder: a case report

**DOI:** 10.3389/fneur.2023.1141835

**Published:** 2023-04-17

**Authors:** Richard Friedrich Radlberger, Alexander Baden Kunz

**Affiliations:** Department of Neurology, Christian Doppler Medical Center, Paracelsus Medical University, Salzburg, Austria

**Keywords:** vitamin D, vitamin B and folic acid, non 24 h sleep–wake disorder, seasonal affective disorder (SAD), case report

## Abstract

We report a case of an emmetropic woman with excessive daytime sleepiness in alternation with insomnia consistent with the diagnosis criteria of a non-24 h sleep–wake disorder. After being refractory to the usual non-pharmacologic and pharmacologic treatment, we detected a deficiency of vitamin B12, vitamin D3, and folic acid. Substitution of these treatments led to a return of a 24 h sleep–wake rhythm though this remained independent from the external light–dark cycle. The question arises whether the vitamin D deficiency could be regarded as an epiphenomenon or whether there is an up-to-date unknown connection to the inner zeitgeber.

## Introduction

The endogenous circadian rhythm in humans persists in constant conditions, i.e., constant darkness, with a period of 24 h. The period of the rhythm in constant conditions is called the innate period and is denoted by the Greek letter *tau*. In healthy humans, *tau* is slightly >24 h (1). A misalignment between extrinsic stimuli (light–dark cycle) and *tau* (2) can cause a free-running state that constantly delays or advances the sleep–wake rhythm. From a clinical perspective, a patient with a circadian rhythm sleep–wake disorder experiences non-restorative sleep, excessive daytime sleepiness, difficulty falling asleep, and/or difficulty maintaining sleep (3). Particularly in sighted individuals, the etiology is not fully understood. In a letter to the editor published in 2022, Kitajima summarized evidence for certain behavioral and environmentally driven causes (4).

Low vitamin D status is quite common especially in the winter months in central Europe (5), and vitamin D deficiency has been associated with impaired sleep (6). Vitamin D seems to have an important role in sleep regulation (7). Vitamin D deficiency increases the risk of sleep disorders and is reported to be associated with sleep difficulties, shorter sleep duration, and nocturnal awakenings (8–10).

In the presented case, the symptoms of a circadian rhythm disorder seem to be connected to a vitamin D deficiency, which is until now neglected in the literature.

## Case report

**Figure 1:**
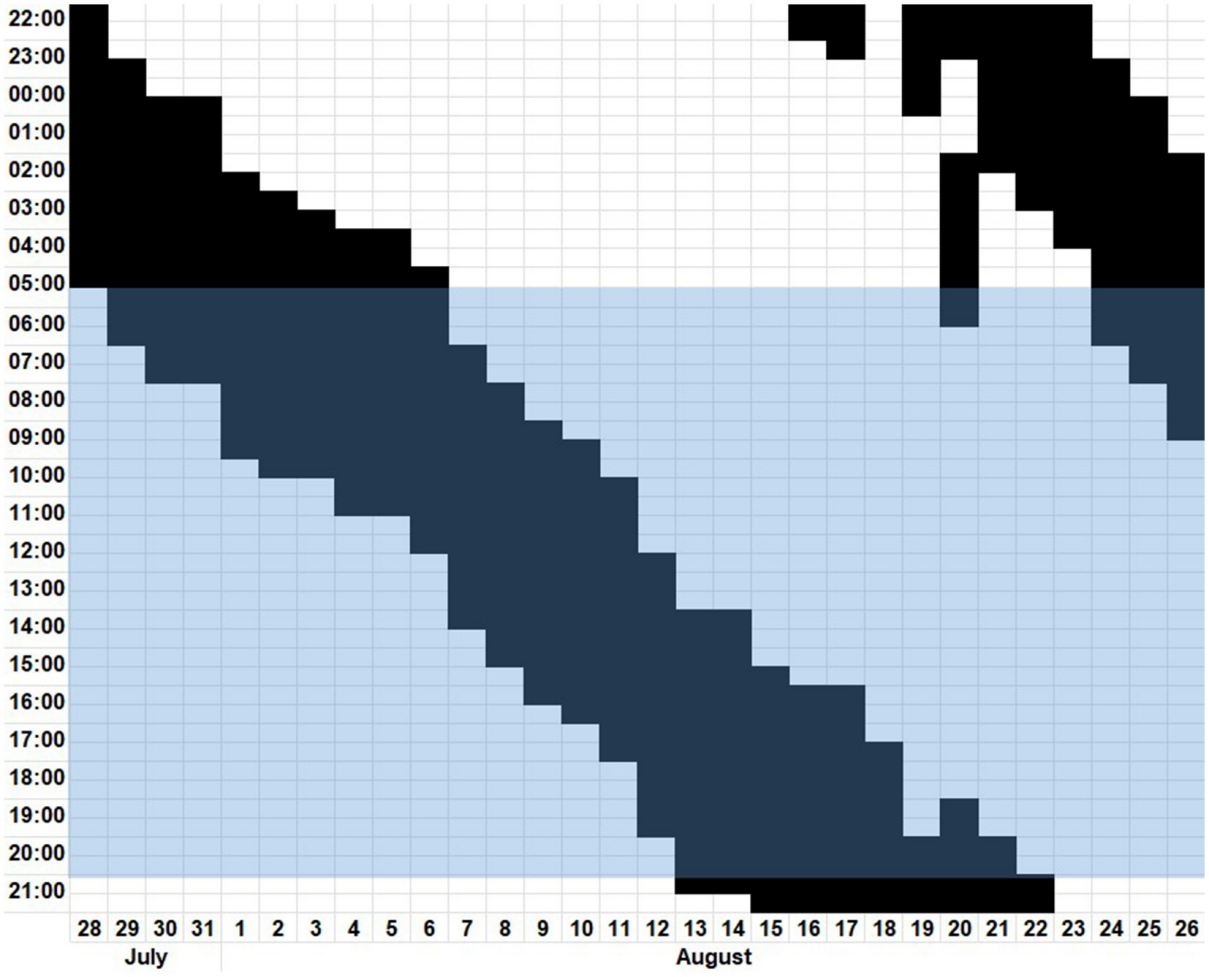
Digitalized patient's sleep diary from 28th July to 26th August depicting the time slept in black and the time awake in white. The blue highlighted block represents the time from sunrise to sunset.

This case is about a 35-year-old woman, working at a kindergarten, with recurring excessive daytime sleepiness over a period of 6 years. She described an aggravation of her symptoms starting a few weeks after the first lockdown due to the COVID-19 pandemic and presented a graph (see Figure 1) displaying her sleep–wake habits. As can be seen in Figure 1, the sleep–wake rhythm, with a *tau* of approximately 25 h, independent of external stimuli, fits the diagnostic criteria (11) of a non-24 h sleep–wake disorder (N24SWD).

At the time of her initial presentation, she had not been taking any medication and had no prior known disease. The family history was negative for sleep disorders.

Initially, we tried various non-pharmacological approaches such as sleep hygiene and prescribed sleep–wake scheduling. We later proceeded to attempt to reset the circadian pacemaker with physical activity, as well as light therapy. Neither method in combination nor in isolation produced any significant effect on the symptoms of our patient. A pharmacological therapy with melatonin was therefore trialed, which also failed to elicit an adequate response.

**Figure 2:**
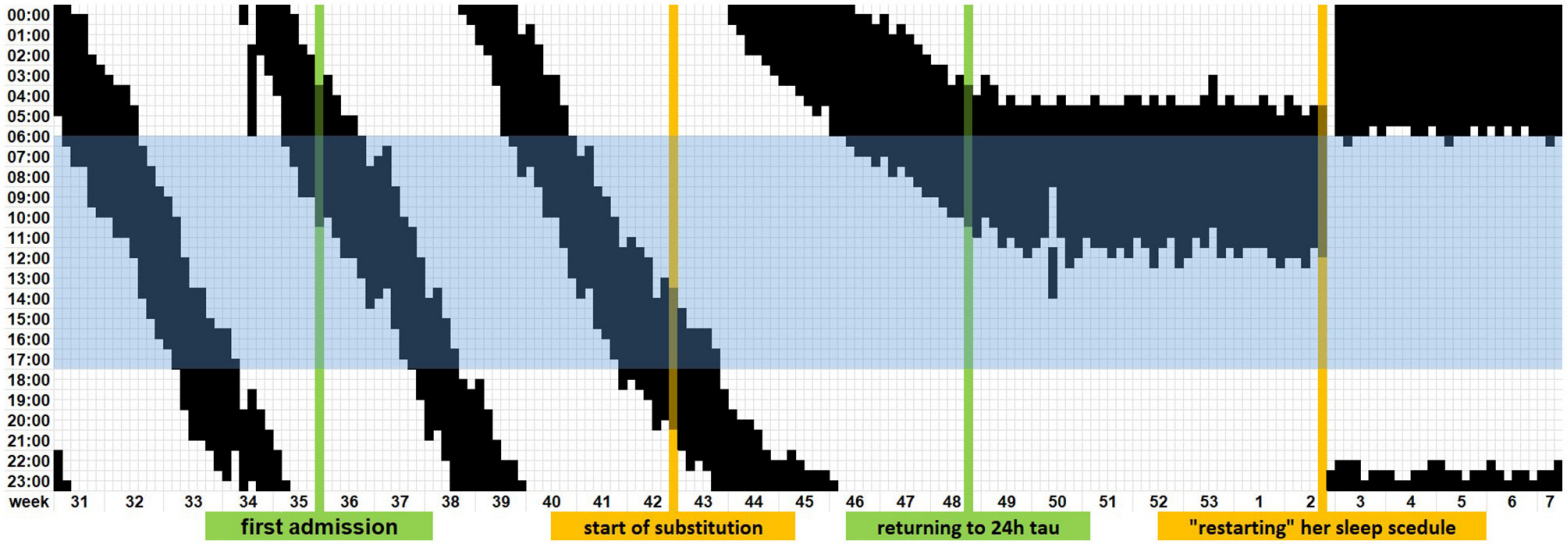
Timeline of the digitalized patient-reported sleep diary until normalization of the sleep–wake rhythm. The blue highlighted block represents regular working hours in two shifts.

At this point, we expanded the laboratory examination to include serum levels of vitamins, thyroid hormones, and trace elements resulting in the detection of insufficient levels of cobalamin (vitamin B12, 189 pg/ml, -10% of lower limit of normal), 25-hydroxy-cholecalciferol (vitamin D3, 7.8 ng/ml, -74% of lower limit of normal), and folic acid (0.9 ng/ml, -71% of lower limit of normal). An additionally conducted polysomnography showed no significant pathologies except for fractioning of sleep. Substitution of the abovementioned deficiencies subsequently resulted in a resynchronization to an almost 24-h rhythm within 1 month. The vitamin and folic acid levels at that time roughly surpassed the lower limit of normal. Even after 5 weeks, her bedtime stayed at 5 a.m. in the morning. With 7 h needed to be well rested, a normal work schedule in a kindergarten starting at noon is rather complicated. Henceforth, we recommended that she stayed awake for one night. The next day she went to bed at her usual time of 11 p.m. and woke up at 6 a.m. without an alarm. Figure 2 displays a graphical summary of this.

However, after cessation of the substitution, a recurrence of symptoms was noted. The patient, therefore, consulted us via e-mail, and we advised her to recommence the substitution of vitamin D with a target level of roughly the upper limit of normal and to “restart” her sleep schedule. In the ensuing weeks, even her preceding repeated daytime sleepiness improved, and she could return to her work as a kindergarten teacher as before. Vitamin levels monitored at the check-ups in our outpatient clinic showed stable values under continuous oral substitution of 11.200 I.E. cholecalciferol per week.

## Discussion

In this case report, we show a possible relationship between the deficiency of vitamin D3, folic acid, and vitamin B12 and an N24SWD. In the literature, only a limited number of cases have been reported that describe the development of an N24SWD in normal-sighted individuals (12). Hence, evidence regarding treatment regimens is restricted. The abovementioned non-pharmacological therapy such as sleep–wake schedule, physical activity, light therapy, as well as pharmacological attempts with melatonin or melatonin agonists are documented in small studies or case reports, on the basis from which a strong recommendation cannot be derived (3).

Earlier case reports (13–15) had already documented the positive effects of vitamin B12 substitution independent of a measured deficiency or typical clinical symptoms and had discussed its possible impacts on the sleep–wake rhythm, as well as on entrainment of the endogenous sleep–wake rhythm to the environment. In contrast to our case, none of the aforementioned cases had described a deficiency of vitamin B12. Unfortunately, the serum levels of methylmalonic acid and homocysteine were not reported in these cases. It would be of interest to know whether correspondingly deviated values had been identified, as in combination, these parameters seem to be more sensitive in depicting a vitamin B deficiency (16). Furthermore, in these cases, vitamin B substitution showed a substantial and lasting effect, which subsided after discontinuation (13). In our case, it was decided to recommence monotherapy substitution for the most profound deficiency, namely, vitamin D, upon the recurrence of symptoms. Interestingly, the substitution of this alone was sufficient to subdue the symptoms.

To the best of our knowledge, this is the first described case of a non-visually impaired patient whose symptoms of an N24SWD seem to correlate to abnormal levels of vitamin D and/or folic acid. Taking into account the connection between vitamin D levels and seasonal affective disorders (SAD) as a form of depression related to climate and seasonal weather changes (17), it is possible that inattentiveness and fatigue could be misunderstood as daytime sleepiness and as a form of non-restorative sleep. Low levels of vitamin D are linked to depression and may also cause sleep disturbances (18). Vitamin D has been shown to have both direct and indirect effects on serotonin and melatonin levels (11). Furthermore, studies have described the involvement of vitamin D in the production pathways of melatonin, the hormone responsible for regulating human circadian rhythms and promoting healthy sleep (7). The treatment with oral melatonin had no effect in our case. What remains unclear is why the symptoms of the patient worsened in the summer. It is possible that social withdrawal and lack of structure throughout the day due to the first COVID-19 lockdown in March 2020 might have been the catalyst for the aggravating symptoms of a preceding N24SWD. Potentially, the subsequently diagnosed vitamin D deficiency might only have been the epiphenomenon without substitution of which our patient would not have been able to recover. Moreover, it is also plausible that she had suffered from dysthymia, which was aggravated due to SAD or decreased vitamin D levels in the context of the COVID policies.

After reaching the lower level of normal for the detected deficiencies, our patient returned to a stable *tau* of approximately 24 h although her sleep cycle was still not in line with the external stimuli. The fact that her sleep–wake rhythm remained independent of the sunlight and other attempts to resynchronize it stands in opposition to the theory that the symptoms are only linked to depression. This is supported by the impact of our pragmatic testing to stay awake for 1 day and henceforth going to bed at her usual time earlier to the aggravation after multiple weeks with hardly any change, resulting in a resetting of her sleep–wake rhythm.

## Limitations

Considering the overlap of the symptoms with SAD, a serotonergic therapy with, for example, trazodone could have been trialed after the initial failed treatment with melatonin.

Therapeutically, we might also have considered intramuscular injections of vitamin B in the setting of a serological vitamin B deficiency (16). However, previous case reports describing the management of neurological manifestations of vitamin B deficiencies (13–15) instead described treatment with oral cobalamin as well as oral substitution proved to be adequately effective in reducing symptoms for the patient in question.

Another limitation is that further laboratory analysis was not carried out. Particularly, in the setting of multiple vitamin deficiencies, it would have been of interest to have additionally analyzed methylmalonic acid and/or homocysteine levels.

Due to the later consultation via e-mail, we were unable to draw a new blood sample and hence could not check whether and how low the vitamin levels had dropped. Pragmatically, we suggested restarting the substitution for the most pronounced deficiency, namely, vitamin D, which was sufficient in returning her *tau* to a 24-h rhythm.

## Conclusion

Previously published material regarding the substantial and long-lasting effect of oral vitamin B12 (13) and the described symptoms, in this case, could not be definitively related to one another. However, at least the marginal impact of vitamin deficiency on the patient's symptoms cannot be excluded. Regarding laboratory investigation, we would suggest that in cases of suspected cobalamin deficiency, homocysteine as well as methylmalonic acid levels should be added to a routine laboratory panel.

Inattentiveness and fatigue as typical symptoms in a SAD (17) could be misunderstood in the context of a prolonged *tau*. These have been described in association with vitamin D metabolism (19) and unsurprisingly were affected by the substitution therapy. The independence of external stimuli as well as the effect of the “restarting” of the sleep–wake rhythm promotes the concept of a connection to the innate period. For the establishment of the pathophysiological concept, further examinations will be needed. Meanwhile, we would consider this as a further treatment option in patients with symptoms of an N24SWD.

## Data availability statement

The original contributions presented in the study are included in the article/supplementary material, further inquiries can be directed to the corresponding author.

## Ethics statement

Written informed consent was obtained from the individual(s) for the publication of any potentially identifiable images or data included in this article.

## Author contributions

RR: drafting the manuscript. AK: critical reading and final approval of the manuscript. Both authors contributed to the article and approved the submitted version.
